# Effect of Intracanal Medications on the Viability of Human Periodontal Ligament‐Derived Mesenchymal Stem Cells

**DOI:** 10.1111/aej.70020

**Published:** 2025-09-16

**Authors:** Arian Braido, Walbert de Andrade Vieira, Bruno Cazotti Pereira, Karina Gonzales Silvério, Paulo Henrique Gabriel, Aline Cristine Gomes Matta, Emerson Alves Martins, Adriana de Jesus Soares

**Affiliations:** ^1^ Department of Restorative Dentistry, Endodontics Division, Piracicaba Dental School Universidade Estadual de Campinas – UNICAMP Piracicaba SP Brazil; ^2^ Departament of Dentistry Centro Universitário das Faculdades Associadas de Ensino – UNIFAE São João da Boa Vista SP Brazil; ^3^ Department of Restorative Sciences University of Alabama at Birmingham, School of Dentistry Birmingham Alabama USA

**Keywords:** cell survival, mesenchymal stem cells, regenerative endodontics, root canal medicaments

## Abstract

This in vitro study evaluated the effects of intracanal medications on the metabolic activity and adhesion of human periodontal ligament‐derived mesenchymal stem cells. Tested groups included Control (without medication), Triple Antibiotic Paste (TAP), Double Antibiotic Paste (DAP), calcium hydroxide with saline, calcium hydroxide with 2% chlorhexidine gel and Bio‐C Temp. After conditioning, roots were stored in phosphate‐buffered saline at 37°C for 21 days. Medications were then removed using 17% EDTA and saline, and dentine samples were used as substrates for cell culture at 3, 5 and 7 days. Metabolic activity was assessed via MTT assay, and adhesion via scanning electron microscopy. None of the medications negatively affected cell viability or adhesion compared to control. TAP showed higher metabolic activity than calcium hydroxide with saline at Day 7. DAP induced earlier cell morphology changes. Bio‐C Temp and chlorhexidine gel showed no significant differences at Day 7, confirming overall biocompatibility.

## 1 Introduction

Regenerative endodontic procedures (REPs), also known as revascularisation or revitalisation, have become increasingly prominent in modern endodontics. These procedures promote continued root development, increasing root length and dentine thickness [1, 2]. During REPs, a blood clot forms inside the root canal, acting as a scaffold for the migration of undifferentiated mesenchymal cells [3, 4]. The goal is to regenerate a tissue similar in structure and function to dentine and pulp, although it does not completely replicate them [3, 4, 5].

Key stem cell sources involved in regeneration include dental pulp stem cells (DPSCs), stem cells from apical papilla stem cells (SCAP), periodontal ligament stem cells (PDLSCs) and bone marrow stem cells (BMSCs) [6, 7]. PDLSCs differentiate into mesenchymal cell lineages to produce cementoblast‐like cells, adipocytes and periodontal ligament fibroblasts that secrete tissue rich in type I collagen [8, 9, 10, 11, 12]. Animal studies have shown that tissues formed within the canal space of immature teeth were characterised as bone, cementum and periodontal ligament‐like tissue [13, 14, 15]. Similar findings have been reported in human immature permanent teeth with necrotic pulp or apical periodontitis after REPs [15, 16, 17].

Intracanal medication is a cornerstone of REP disinfection and is crucial to treatment success [18]. These medications must eliminate bacteria while maintaining an environment that supports cell survival and proliferation [19, 20].

Triple antibiotic paste (TAP), composed of metronidazole, minocycline and ciprofloxacin, has been widely used due to its effectiveness in infection control and tissue formation [21]. However, minocycline may cause crown discoloration [22, 23]. As alternatives, double antibiotic paste (DAP), which omits minocycline, and calcium hydroxide‐based pastes have been proposed [22, 24]. The combination of calcium hydroxide and 2% chlorhexidine gel has shown high success rates after 21 days in the root canal, benefiting from the antimicrobial synergy of both agents [3, 22, 25].

Recently, calcium silicate‐based medications such as Bio‐C Temp (Angelus, Brazil) have gained attention [26, 27]. This ready‐to‐use paste contains tricalcium silicate, dicalcium silicate, tricalcium aluminate, calcium oxide and other components. It has a resin‐based vehicle that prevents hydration and setting, which may influence ion release and biological response [28]. Bio‐C Temp is indicated as an intracanal medication, eliminating the need for frequent changes compared to calcium hydroxide‐based pastes, and has been suggested for cases of incomplete root formation, resorptions, perforations, pulpotomy and regenerative procedures [26]. It has demonstrated high biocompatibility and antimicrobial activity in vitro [25], although human dental pulp cells showed dose‐ and time‐dependent cytotoxicity [26], while in vivo studies reported biocompatibility and no systemic toxicity [25].

Given its bioactive properties, Bio‐C Temp appears promising for REPs [26, 27]. However, no studies have investigated its indirect effects on PDLSCs.

Therefore, this study aims to evaluate the indirect effects of Bio‐C Temp compared to REPs medications on the metabolic activity and adhesion of undifferentiated mesenchymal cells derived from the human periodontal ligament (hPDLSCs) on intraradicular dentine.

## 2 Materials and Methods

**FIGURE 1:**
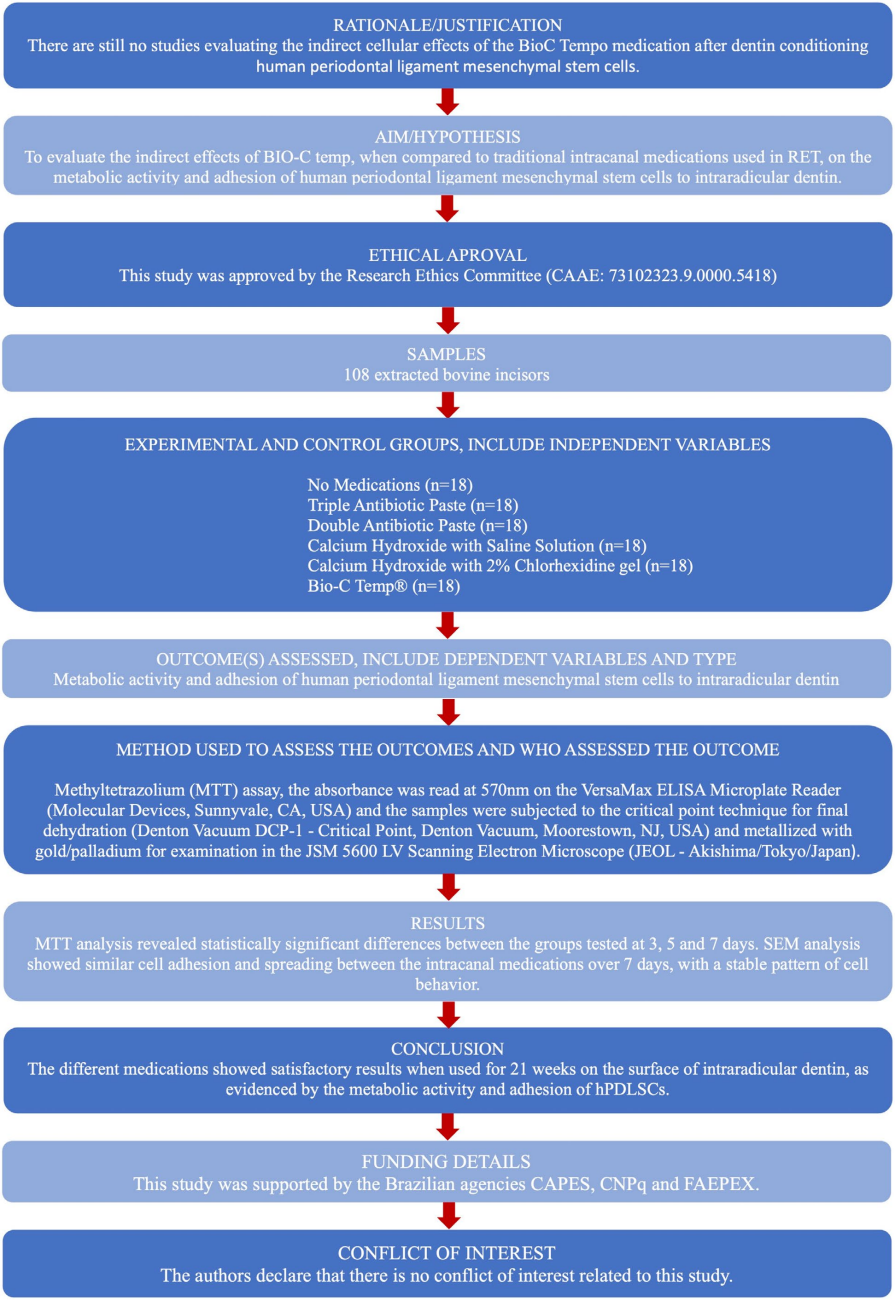
PRILE 2021 flowchart used to guide study design and reporting.

This study followed the PRILE 2021 guidelines for laboratory studies in endodontology [29] and received approval from the institutional research ethics committee (CAAE: 73102323.9.0000.5418). The PRILE 2021 flowchart is presented in Figure 1.

### 2.1 Sample Size Calculation

The required sample size was calculated using a one‐way ANOVA test (*F* test family), with an effect size of 2.0800, alpha of 0.05 and beta of 0.01. Based on these parameters, 108 specimens were included: 15 per group for metabolic activity and 3 for descriptive adhesion analysis.

### 2.2 Cell Population

Three populations of hPDLSCs (Biobanco Periocells B041, FOP‐UNICAMP) were previously characterised [28]. Periodontal ligament tissue was enzymatically digested using collagenase/dispase (Gibco), centrifuged, and the resulting cell pellet was cultured in DMEM supplemented with 10% FBS, 100 μg/mL streptomycin and 100 U/mL penicillin at 37°C, 5% CO_2_ and 98% humidity until confluence. Cells were isolated using magnetic separation (MACS, Miltenyi Biotec) and confirmed to exhibit a mesenchymal phenotype by flow cytometry, expressing STRO‐1, CD105 and CD166 markers and negative surface markers to CD34 and CD45 [30, 31]. Cells from passages 5–7 were used in triplicate for each experiment.

### 2.3 Specimen Selection and Preparation

**FIGURE 2:**
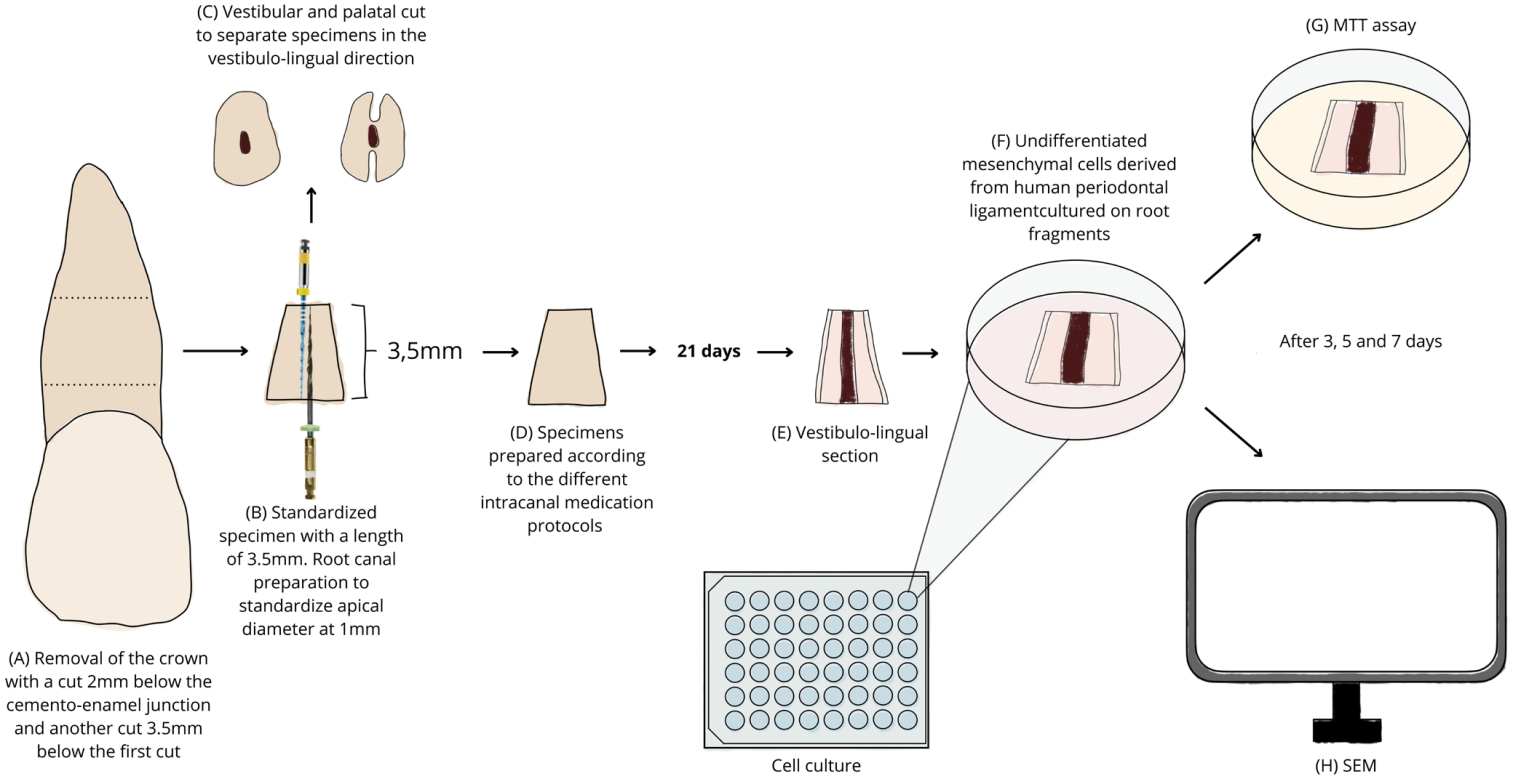
Specimen preparation, cell culture and analyses applied. (A) Crowns were removed 2 mm below the cementoenamel junction. (B) Root canals were standardised to a minimum diameter of 1 mm. (C) Samples were prepared for cleaving to expose the intraradicular dentine surface. (D) Specimens were medicated for 21 days. (E) Sample after medication removal and cleavage. (F) hPDLSCs cultured on dentine for 3, 5 and 7 days. (G) Specimen transferred for MTT analysis. (H) Specimen prepared for SEM.

Under irrigation, 54 bovine roots were sectioned to 3.5 mm using a precision cutter (ISOMET, Buehler). Crowns were removed 2 mm below the cementoenamel junction (Figure 2A). Root canals were standardised to a minimum diameter of 1 mm (Figure 2B). Apical regions were sealed with composite resin to prevent leakage of irrigants and medications. The specimens were stored in 1.5 mL PBS and autoclaved at 121°C.

### 2.4 Irrigation Protocol and Intracanal Medications

Specimens were irrigated with 20 mL of 2.5% sodium hypochlorite, followed by 20 mL of saline solution and 10 mL of 17% EDTA at a flow rate of 0.06 mL/s for 5 min. Irrigants were aspirated using sterile metal cannulas. Canals were dried with capillary tips (Ultradent) and sterile absorbent paper points.

Specimens were divided into three groups according to the cell population, then randomly assigned to six experimental groups (www.randomizer.org): Negative Control (G‐C), Triple Antibiotic Paste (G‐TAP), Double Antibiotic Paste (G‐DAP), Calcium Hydroxide + Saline (G‐HC + SS), Calcium Hydroxide + 2% Chlorhexidine Gel (G‐HC + CHX) and Bio‐C Temp (G‐BIOC).

G‐TAP, G‐DAP, G‐HC + SS and G‐HC + CHX were prepared on sterile glass plates and inserted using syringes. G‐BIO‐C was applied per the manufacturer's instructions using its applicator. After placement, roots were sealed with Z250 XT composite resin (3 M) and stored in PBS (pH 7.4) at 37°C for 21 days.

Afterward, intracanal medications were removed using 20 mL of 17% EDTA at a flow rate of 0.06 mL/s for 5 min, followed by 10 mL of saline solution. Canals were dried, resin seals were removed, and the roots were cleaved buccolingually to yield 108 samples for cell culture.

### 2.5 Methylthiazole Tetrazolium Assay (MTT)

Fifteen conditioned specimens per experimental group (five per evaluation time point) were used to assess the effects of intracanal medications on cellular metabolic activity. Additionally, the control group (Plate) was included, in which hPDLSCs were cultured directly in standard 48‐well culture plates, without dentine specimens or exposure to intracanal medications. The Plate group also consisted of five wells per time point (3, 5 and 7 days). This group was used to control cell growth.

For experimental groups, hPDLSCs (2 × 10^4^ cells/specimen) were seeded on conditioned dentine surfaces in 48‐well plates with 400 μL of DMEM supplemented with 2% FBS and 1% Pen/Strep. The Plate group received the same number of cells and medium volume under identical incubation conditions (37°C, 5% CO_2_).

At each time point, dentine specimens (*n* = 5 per group) were transferred to 24‐well plates, and the MTT assay was performed using 3‐(4,5‐dimethylthiazol‐2‐yl)‐2,5‐diphenyl tetrazolium bromide (Sigma, #M2128). After 4 h of incubation, the medium was removed, and 400 μL of 100% ethanol was added. After 15 min, 100 μL of the resulting solution was transferred to a 96‐well plate in triplicate, and absorbance was measured at 570 nm using a microplate reader (VersaMax ELISA, Molecular Devices).

### 2.6 Scanning Electron Microscopy (SEM)

Three additional specimens per group were used for SEM analysis to assess cell adhesion and morphology. hPDLSCs (2 × 10^4^ cells/specimen) were cultured on dentine surfaces at 5% CO_2_ and 37°C for 3, 5 or 7 days. After incubation, samples were washed with PBS, fixed in glutaraldehyde for 48 h, washed, dehydrated in graded ethanol (50%–100%, 20 min each) and subjected to critical‐point drying (Denton Vacuum DCP‐1). Samples were mounted on stubs, sputter‐coated with gold–palladium and examined under a scanning electron microscope (JSM 5600 LV, JEOL). Four images per sample were captured at 250× and 500× magnification.

### 2.7 Statistical Analysis

The data were analysed using R software (version 4.4.3), with support from the ‘stats’, ‘RVAideMemoire’, ‘car’, ‘rstatix’ and ‘ggplot2’ packages. Absorbance values from each experimental group were normalised as percentages relative to the control plate containing only cells. The Shapiro–Wilk and Levene tests were used to assess data normality and homogeneity of variances, respectively. As the data did not follow a normal distribution and showed no homoscedasticity (*p* < 0.05), group comparisons were performed using the Kruskal–Wallis test followed by Dunn's post hoc test with Holm–Bonferroni correction. The significance level was set at 5% for all analyses.

## 3 Results

### 3.1 Methylthiazole tetrazolium assay (MTT)

Mitochondrial metabolic activity of hPDLSCs was evaluated at 3, 5 and 7 days. The Plate group, in which cells were cultured directly on plastic without dentine, served as an additional control for comparison.

**FIGURE 3:**
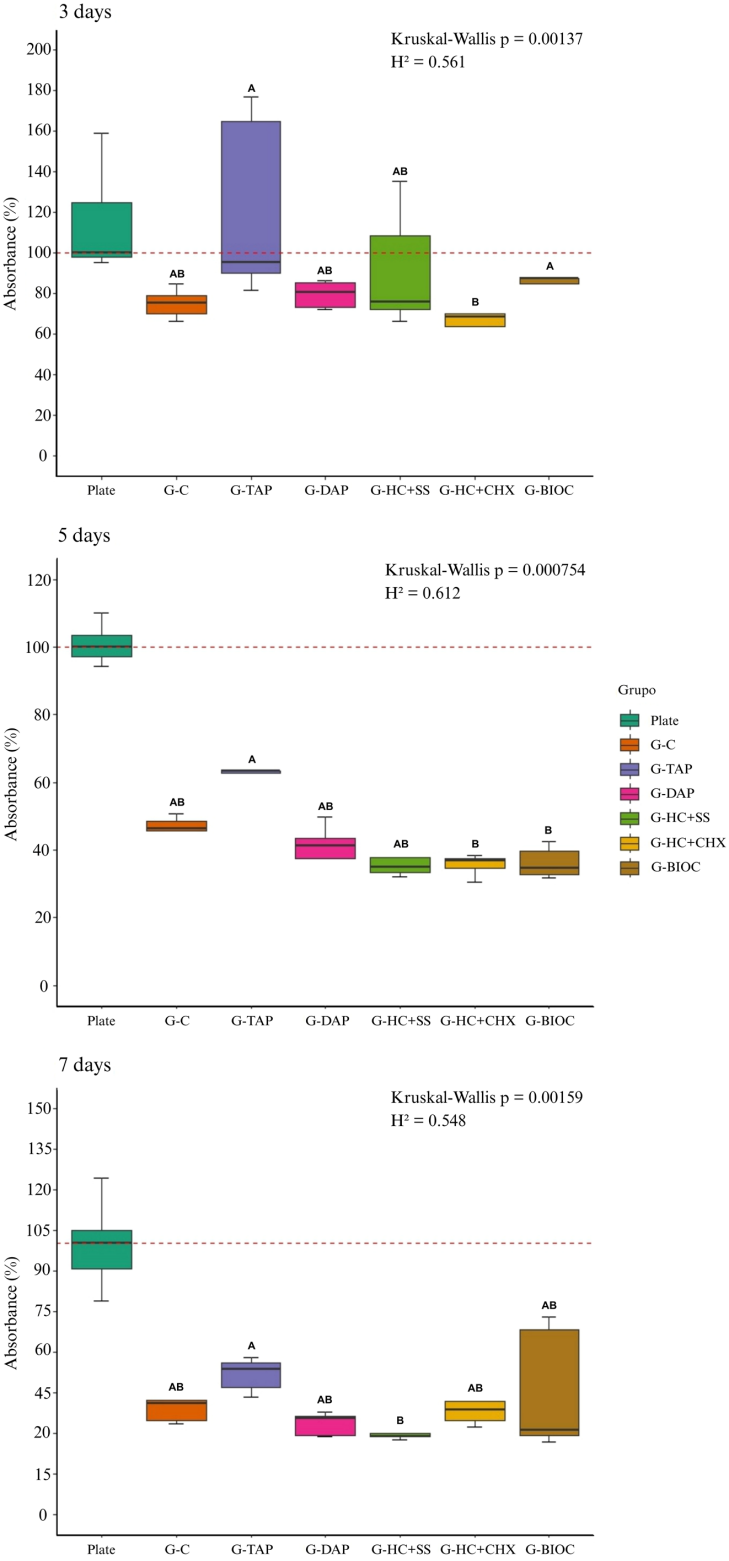
Mitochondrial metabolic activity of hPDLSCs in response to six intracanal medications and a plastic plate control (Plate), assessed using the MTT assay at 3, 5 and 7 days. Cells in the Plate group were cultured directly on standard plastic wells without dentine or medication. Each graph shows median values and interquartile ranges (25%–75%). Different capital letters indicate statistically significant differences between groups on the same day, while the same capital letters indicate no significant difference (*p* < 0.05; Kruskal–Wallis with Dunn's post hoc and Holm–Bonferroni correction).

At Day 3, G‐BIOC and G‐TAP exhibited significantly higher metabolic activity compared with G‐HC + CHX (*p* < 0.05). At Day 5, no significant difference was observed between G‐HC + CHX and G‐BIOC, although G‐TAP showed higher values than both groups (*p* < 0.05). At Day 7, G‐BIOC did not show significant differences compared with any of the studied groups, while G‐TAP exhibited significantly higher metabolic activity than G‐HC + SS (*p* < 0.05). These differences had moderate to large effect sizes (Figure 3).

When compared with the REP protocol without medication (G‐C), all protocols that included medicament application maintained cell viability above 70%, according to ISO 10993‐5 criteria, indicating that the use of these medications did not induce cytotoxicity.

### 3.2 Scanning Electron Microscopy (SEM)

**FIGURE 4:**
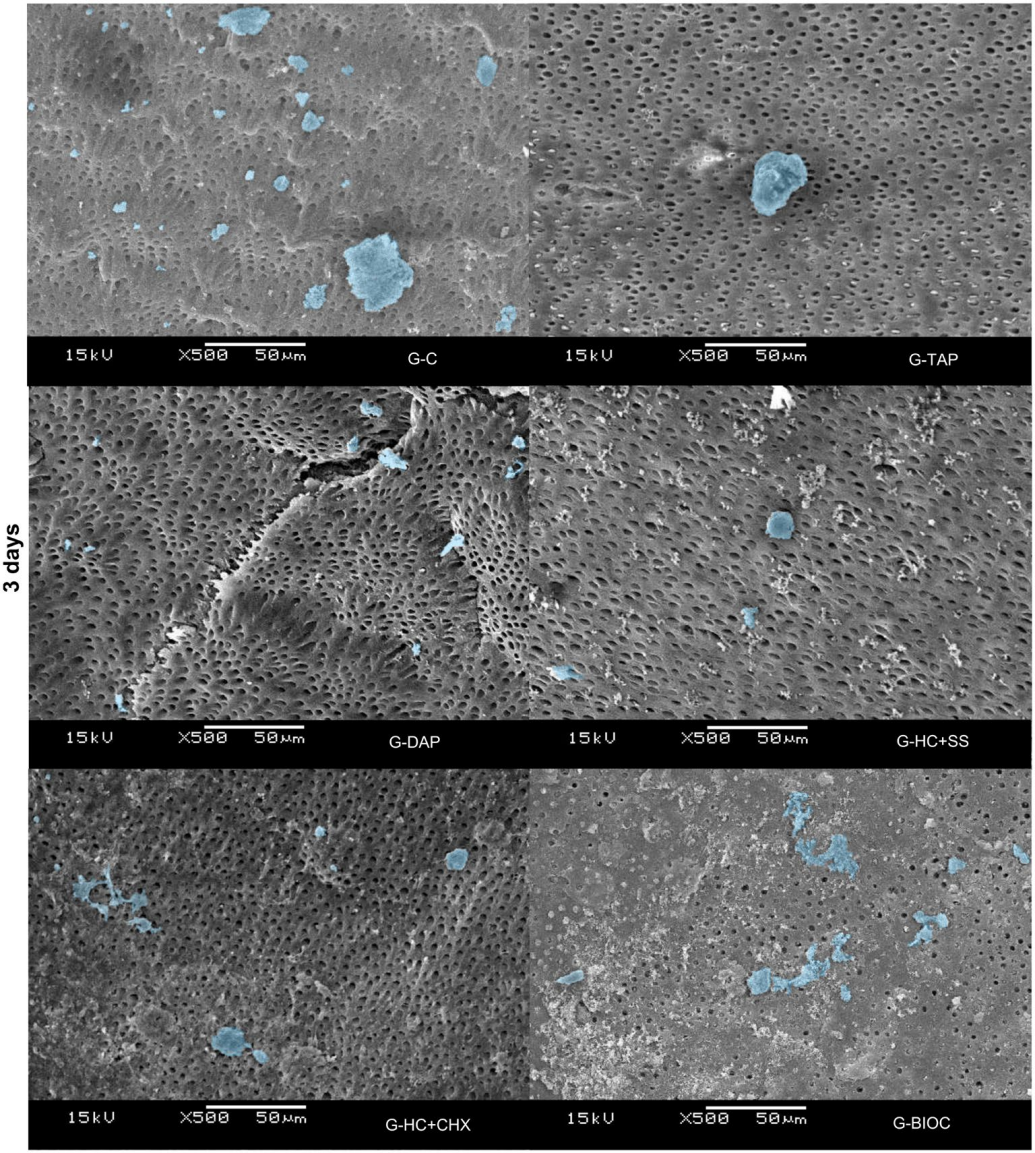
SEM images of hPDLSCs cultured on dentin treated with different intracanal medications at Day 3. Cells are highlighted in blue. Representative images are shown at 500× magnification.

**FIGURE 5:**
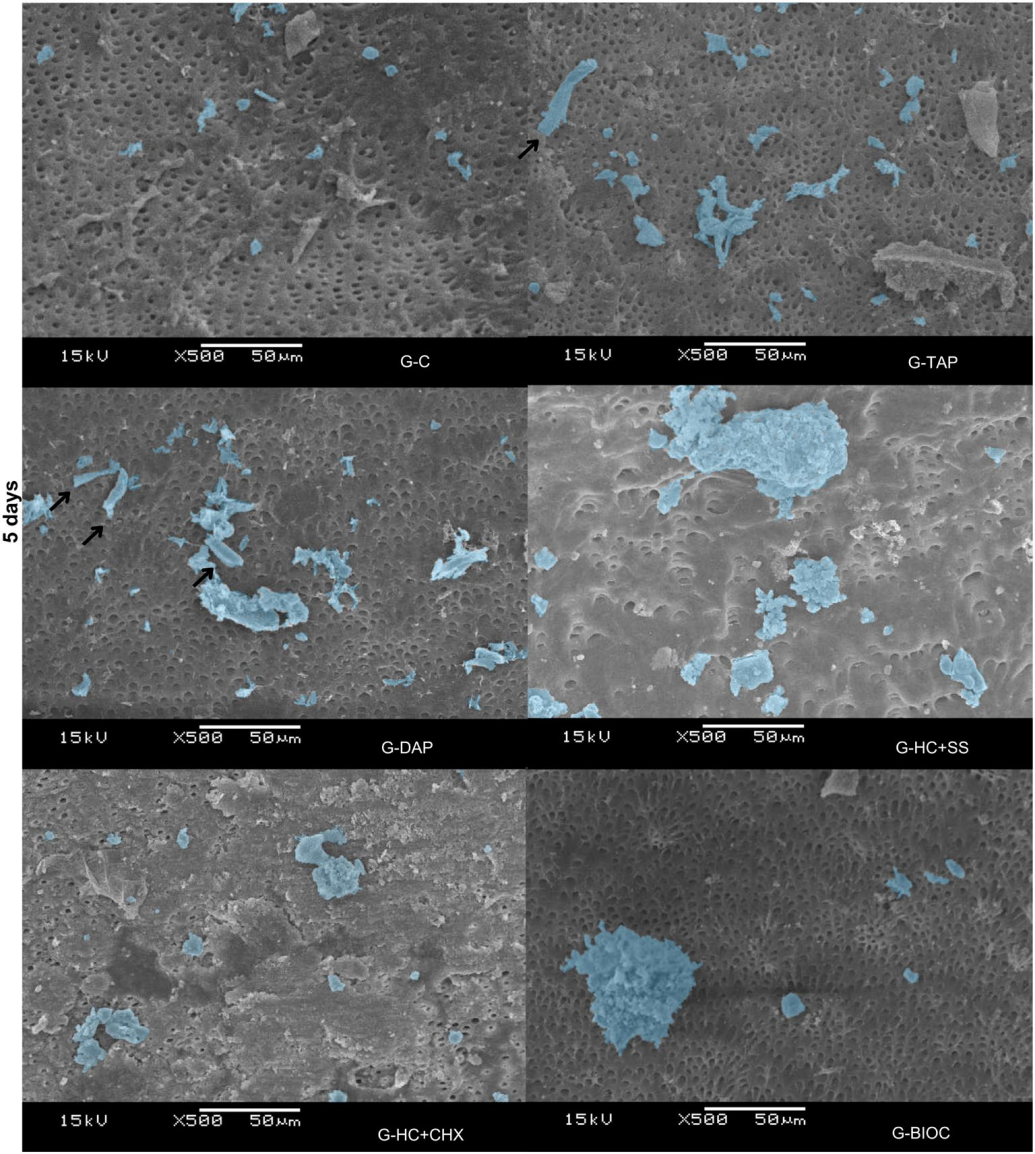
SEM images of hPDLSCs cultured on dentine treated with different intracanal medications at Day 5. Cells are highlighted in blue. Arrows indicate adhered cells on the dentine surface, evidencing cell attachment and morphological adaptation. All groups exhibited a similar pattern of cell adhesion. Representative images are shown at 500× magnification.

**FIGURE 6:**
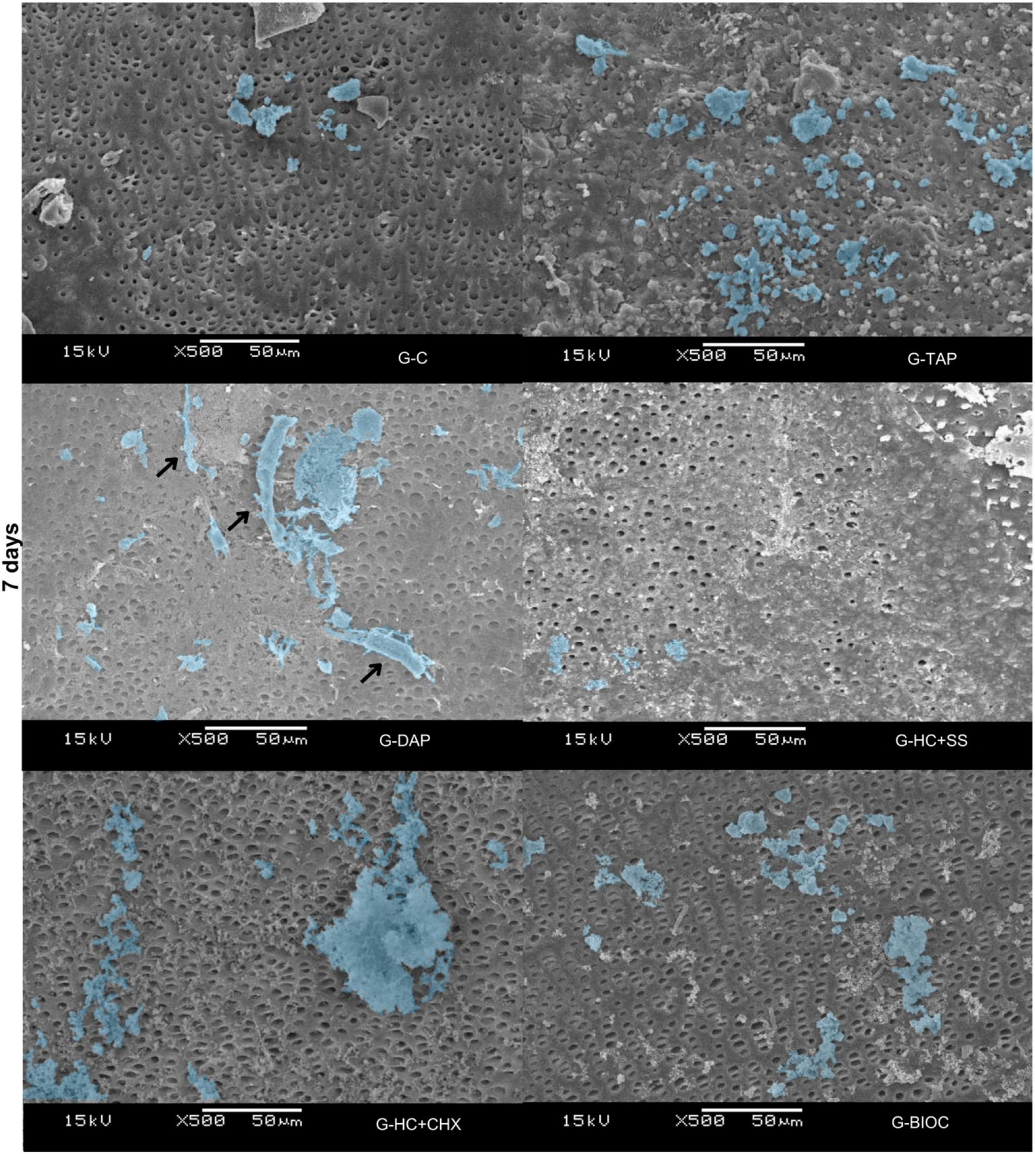
SEM images of hPDLSCs cultured on dentine treated with different intracanal medications at Day 7. Cells are highlighted in blue. Arrows indicate adhered cells on the dentine surface, evidencing cell attachment and morphological adaptation. The G‐DAP group displayed more elongated cell morphology, indicative of enhanced adhesion. Representative images are shown at 500× magnification.

SEM analysis at Days 3, 5 and 7 demonstrated that hPDLSCs exhibited comparable adhesion and spreading on dentine surfaces across all experimental groups. Notably, the G‐DAP group presented a more elongated cell morphology at Days 5 and 7, indicative of enhanced and accelerated cellular attachment relative to the other groups (Figures 4, 5, 6).

## 4 Discussion

This study evaluated the indirect effects of Bio‐C Temp, in comparison with intracanal medications traditionally used in REPs, on the metabolic activity and adhesion of hPDLSCs in intraradicular dentine. The results showed that, in the analysis of mitochondrial activity using the MTT assay, by the final time point of the experiment, Bio‐C Temp showed no statistically significant difference in cellular metabolic activity when compared to the other intracanal medications, demonstrating a similar level of cell viability to those already used in REPs. Regarding the adhesion analysis using SEM, all groups exhibited surface characteristics indicative of cell attachment; however, the DAP showed morphological features suggestive of faster and more favourable cell adhesion compared to the other medications.

Despite the advances in REPs, only Ghabraei et al. [32] have evaluated the effects of intracanal medications directly on hPDLSCs. Volponi et al. [8] highlighted that these cells have the ability to differentiate into mesenchymal lineages, giving rise to cementoblast‐like cells, adipocytes and periodontal ligament fibroblasts that secrete tissue rich in type I collagen, both in in vitro and in vivo experiments. They may have effects similar to those of apical papilla stem cells [33]. Studies such as those by Althumairy et al. [34], Park et al. [35] and Riaz et al. [19] used similar methodologies to culture different cells on the surface of intraradicular dentine. However, none of these authors evaluated the different medications (TAP, DAP, calcium hydroxide with saline, 2% chlorhexidine and Bio‐C Temp) on hPDLSCs.

In this study, the cellular metabolic activity of G‐TAP increased significantly compared to G‐HC + CHX on Days 3 and 5, G‐BIOC on Day 5 and G‐HC + SS on day 7. Previous studies confirm that the results of TAP were better than those of calcium hydroxide when evaluated by the MTT assay carried out at 3 and 7 days using dental pulp cells (DPSCs) in the treated dentine of human teeth [19]. These results can be explained by the fact that TAP has an acidic pH (2.9), while DAP has a slightly higher pH (3.4). The acidic environment of these pastes promotes dentine demineralisation, dissolving mineral components such as calcium and phosphate from the dentine matrix. Minocycline, present in TAP, has a high chelating capacity, removing calcium ions from the mineralised dentine matrix. This effect is amplified by the presence of irrigants such as EDTA, which also act as demineralising agents. The removal of the mineral layer by the action of pastes exposes the collagen fibres of dentine, which are an essential part of the organic matrix. This exposure is an important step in regenerative treatments, as it allows the release of growth factors stored in the dentine matrix [36, 37]. However, antibiotic medication has been described by some authors as causing tooth discolouration due to the presence of minocycline in its composition [22, 23].

G‐BIOC showed statistically significant differences compared to the G‐HC + CHX group on Day 3. On Day 5, the G‐TAP group exhibited better outcomes than the bioceramic medication, while on Day 7, no significant differences were observed between any of the groups. The results found on Day 7 corroborate Silveira et al. [38], who evaluated the cellular metabolic activity of Bio‐C Temp using the MTT assay on a human osteosarcoma cell line (Saos‐2) at 24 and 72 h with different dilutions of the medication. The results showed that, in terms of cellular metabolic activity, Bio‐C Temp did not show any statistical difference from the control group. Similar results were found by Guerreiro et al. [26], who evaluated the cellular metabolism of Saos‐2 at 1, 3 and 7 days with different dilutions of Bio‐C Temp, revealing no cytotoxic effects at the highest dilutions. However, the methodology used by the authors did not assess the residual effect of Bio‐C Temp on intraradicular dentine in hPDLSCs. Furthermore, Bio‐C Temp also has the potential to alter colour due to calcium tungstate [23].

Previous studies indicate that the high alkalinity of Bio‐C Temp favours osteoblastic activity and creates a microenvironment conducive to mineral deposition, which is essential for the regeneration of periapical tissue [26, 38]. Bio‐C Temp has shown significant antimicrobial capacity, especially against *Enterococcus faecalis* , which is frequently associated with endodontic infections [35]. Guerreiro et al. [26] state that the medication had a significantly lower antimicrobial capacity compared to other calcium hydroxide‐based medications, such as Calen and UltraCal. This difference can be attributed to the lower formation of calcium hydroxide molecules during the hydration reaction of Bio‐C Temp, which affects its antimicrobial activity [25]. Despite this, Bio‐C Temp showed greater alkaline phosphatase activity in osteoblastic cells, indicating a positive potential in terms of biocompatibility and stimulation of mineralisation. This could be useful in specific clinical contexts aimed at tissue repair and regeneration, since the equilibrium between antimicrobial activity and maintaining cell viability is crucial to the success of REPs [26, 38]. These findings highlight the importance of considering Bio‐C Temp as a viable alternative in REPs, especially in cases of teeth with incomplete root formation or pulp necrosis.

The calcium hydroxide‐based medication combined with 2% chlorhexidine gel exhibited significantly lower cellular metabolic activity compared to both the TAP and Bio‐C Temp on Day 3. By Day 5, this difference remained significant only in comparison to the TAP, with no statistical difference observed between the calcium hydroxide group and Bio‐C Temp. In the evaluation on Day 7, G‐HC + CHX showed an increase in mitochondrial metabolic activity, with no statistical difference from the other drugs. This can be explained by an immediate transient inhibitory effect of 2% chlorhexidine gel on hPDLSCs. Trevino et al. [39] suggested that the substantivity of CHX may influence the ability of cells to attach to the extracellular dentine matrix, indirectly leading to a loss of cell viability. Saberi et al. [40] found that the addition of chlorhexidine to calcium hydroxide or TAP increased cytotoxicity. In contrast, the study by Ghabraei et al. [32] evaluated the effect of different intracanal medications on hPDLSCs, including combinations of chlorhexidine with MTA and with calcium hydroxide. The authors stated that in the calcium hydroxide with chlorhexidine group, the combination likely reduced toxic effects, as calcium hydroxide modulates the pH of the medium and can reduce the reactivity of CHX, explaining the better results. Calcium hydroxide medication with 2% chlorhexidine gel has already been used as an intracanal medication in REPs [2, 21, 25, 41]. This combination has also shown satisfactory results in terms of reducing viable bacteria, increasing root canal thickness, and promoting apical closure of immature permanent teeth [21, 22].

About G‐C, the different medications evaluated showed no statistically significant difference at any of the times evaluated, suggesting that final irrigation with 17% EDTA may have influenced metabolic activity and cell adhesion in this study. EDTA is known to release essential growth factors from dentine, which help stem cell migration and proliferation. Furthermore, the use of EDTA as a final irrigant in REPs may favour proliferation and migration as it reduces cell cytotoxicity [2, 24, 42].

Representative SEM images were used to evaluate cell adhesion in the different experimental groups. The results showed the presence of cells in all groups, corroborating the data obtained by the MTT cell viability assay. Notably, the G‐DAP group displayed more elongated cell morphology at 5 and 7 days, indicative of enhanced adhesion, which was not observed in the other experimental groups.

The selection of intracanal medications must balance antimicrobial efficacy and biocompatibility. The results reinforce that the use of TAP and DAP is effective, but alternatives such as Bio‐C Temp and combinations of calcium hydroxide and chlorhexidine can be less aggressive and present clinical advantages. Irrigation with EDTA plays a central role in REPs, maximising cell adhesion regardless of the intracanal medication used. The authors acknowledge that the study was carried out in vitro using bovine dentine, which may not fully reflect the results in human teeth. Further studies are needed to evaluate the clinical effects of intracanal medications, especially Bio‐C Temp, in REPs and to explore the interaction between mesenchymal cells and dentine treated with different concentrations of medications.

## 5 Conclusion

In this study, when the REP conditioning protocol was applied to dentine, both the presence and absence of intracanal medication resulted in satisfactory biocompatibility with hPDLSCs. At Day 7, TAP demonstrated superior outcomes compared to calcium hydroxide with saline. SEM analysis showed that DAP induced earlier morphological changes in the cells at Days 5 and 7, indicating enhanced adhesion. Bio‐C Temp did not exhibit any statistically significant differences compared to the other tested medications at the final experimental time point.

## Author Contributions

Conceptualization: Arian Braido, Walbert de Andrade Vieira, Aline Cristine Gomes Matta, Adriana de Jesus Soares. Methodology: Arian Braido, Walbert de Andrade Vieira, Karina Gonzales Silvério, Emerson Alves Martins. Formal Analysis: Arian Braido, Walbert de Andrade Vieira, Aline Cristine Gomes Matta, Adriana de Jesus Soares. Investigation: Arian Braido, Bruno Cazotti Pereira, Paulo Henrique Gabriel. Data Curation: Arian Braido, Emerson Alves Martins. Writing – original draft: Arian Braido. Writing – review and editing: Arian Braido, Walbert de Andrade Vieira, Aline Cristine Gomes Matta, Adriana de Jesus Soares.

## Ethics Statement

This study was approved by the Ethic Committee of the Piracicaba Dental School (Protocol number 73102323.9.0000.5418).

## Conflicts of Interest

The authors declare no conflicts of interest.

## Data Availability

The data that support the findings of this study are available from the corresponding author upon reasonable request.
